# Comprehensive bioinformatics analysis of the common mechanism of atherosclerosis and atrial fibrillation: emphasizing mitochondrial metabolic disorder and immune inflammation

**DOI:** 10.3389/fmolb.2025.1595048

**Published:** 2025-06-18

**Authors:** Rui Dai, Xiaotong Lei, Xiaojun Liu, Chen Bian

**Affiliations:** ^1^ Department of Cardiology, Xuzhou First People’s Hospital (The Affiliated Xuzhou Municipal Hospital of Xuzhou Medical University), Xuzhou, China; ^2^ Department of Nephrology, Xuzhou First People’s Hospital (The Affiliated Xuzhou Municipal Hospital of Xuzhou Medical University), Xuzhou, China

**Keywords:** atherosclerosis, atrial fibrillation, bioinformatics analysis, mitochondria metabolism, immune inflammation, machine learning

## Abstract

**Introduction:**

Atherosclerosis (AS) and atrial fibrillation (AF) are clinically intertwined cardiovascular disorders, yet their shared pathophysiological mechanisms remain poorly understood. Mitochondrial dysfunction and immune dysregulation have been implicated separately in both diseases, but their potential crosstalk in driving comorbidity is unexplored. This study aims to uncover common molecular pathways linking AS and AF, focusing on mitochondrial metabolism and immune response, and to identify diagnostic biomarkers using integrative bioinformatics approaches.

**Methods:**

Transcriptomic datasets of AS and AF were analyzed through GO and KEGG enrichment to identify shared biological themes. WWGCNA prioritized 35 mitochondrial genes associated with both diseases. Three machine learning algorithms (LASSO, SVM, and random forest) were applied to screen hub genes, followed by validation in independent datasets. Immune infiltration and single-cell transcriptomic analyses were conducted to assess immune-microenvironment interactions and gene expression at cellular resolution.

**Results:**

GO/KEGG analyses revealed dominant enrichment in mitochondrial oxidative phosphorylation and immune-inflammatory pathways for AS and AF. WGCNA identified 35 mitochondrial hub genes, with MRPS23 and CASP8 emerging as key candidates via machine learning. MRPS23 showed significant downregulation in AS tissues and cellular heterogeneity in scRNA-seq (single-cell RNA sequencing), while both genes exhibited strong correlations with immune cell subsets in both diseases.

**Conclusion:**

This study establishes MRPS23 as a novel biomarker bridging mitochondrial dysfunction and immune dysregulation in AS and AF comorbidity. Its decline in AS suggests a potential role in mitochondrial ribosomal integrity loss driving metabolic stress, while immune-microenvironment interactions highlight its pleiotropic impacts on inflammatory cascades. These findings advance the “mitochondria-immune axis” paradigm for cardiovascular comorbidity, offering MRPS23 as a dual-disease therapeutic target. Further validation in preclinical models and clinical cohorts is needed to translate these insights into diagnostic or therapeutic applications.

## 1 Introduction

Atherosclerosis (AS) is a chronic, progressive, inflammatory vascular disease that mainly affects the medium and large arteries of the body, such as the coronary, carotid and femoral arteries. The condition is characterized by the gradual accumulation of lipids, inflammatory cells, calcification and other substances within the walls of the arteries to form plaques, which can gradually increase in size and harden, leading to thickening of the artery walls and narrowing of the blood flow lumen, thereby reducing or blocking blood flow to organs and tissues (Ross, 1999). Atrial fibrillation (AF) is the most prevalent clinical arrhythmia, and its prevalence is on the rise due to the aging of the population (Lippi et al., 2021). On the one hand, AF is associated with an increased risk of stroke, cardiovascular morbidity, and mortality (Sagris et al., 2021). On the other hand, AS, which is frequently situated within the tunica intima of numerous medium-sized and large arteries, represents a significant contributor to cardiovascular disease, encompassing conditions such as stroke, heart failure, and others (Frostegård, 2013).

Atherosclerosis and atrial fibrillation are two diseases that are closely linked. It has been demonstrated that individuals with carotid plaque formation, atherosclerosis, and even stenosis in AF exhibit a markedly elevated risk of adverse cardiovascular and cerebrovascular outcomes (Wang et al., 2019). In addition, patients with carotid atherosclerosis had nearly twice the rate of ischemic stroke (IS) recurrence compared with nonsignificant carotid stenosis, and concomitant atherosclerosis in patients with atrial fibrillation was also an independent risk factor for long-term stroke recurrence and 30-day mortality (Lehtola et al., 2017). The association between carotid atherosclerosis and the risk of ischemic stroke (IS) and transient ischemic attack (TIA) in patients with AF was also confirmed in a study that utilized available data (Noubiap et al., 2021). A study from Korea suggests that carotid atherosclerosis is an established risk factor for stroke in patients with AF, and that it may improve stroke risk prediction in patients with AF (Cho et al., 2021). Of notes, evidences from meta-analysis suggest that the use of statins, the most common clinical anti-atherosclerotic agents, reduces mortality in patients with AF, which may indicate that atherosclerosis has a detrimental effect on IS in patients with AF (Eun et al., 2020). In summary, the co-occurrence of AF and AS in clinical practice is associated with an increased risk of mortality. Therefore, it is of clinical importance to investigate the underlying common mechanisms to AS and AF comorbidities at the genetic level and to explore promising therapeutic targets.

**FIGURE 1:**
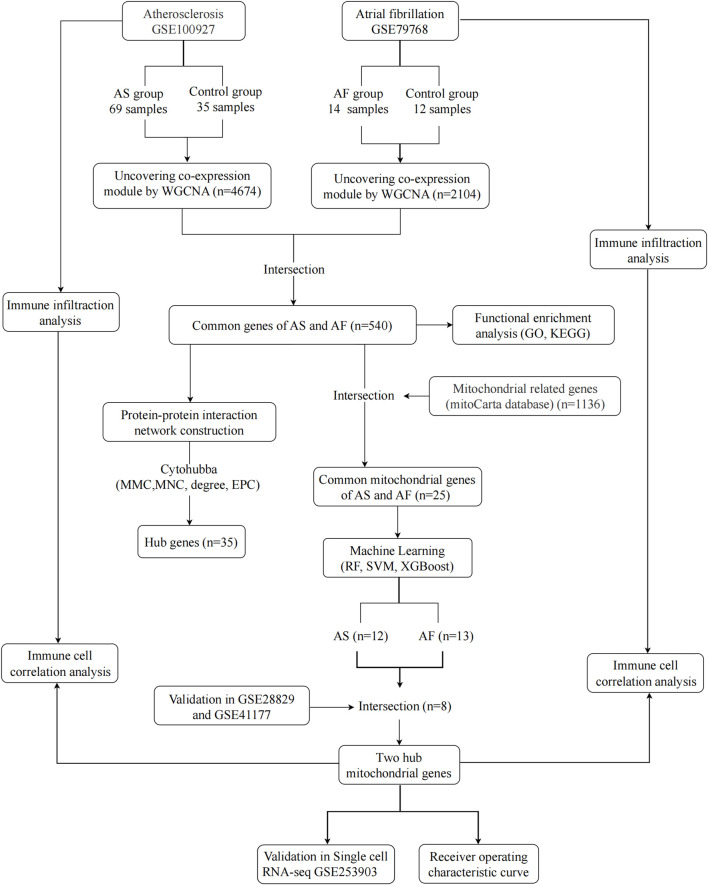
Research Flowchart. GSE, gene expression omnibus series; AS, atherosclerosis; AF, atrial fibrillation; WGCNA, weighted gene co-expression network analysis; GO, gene ontology; KEGG, kyoto encyclopedia of genes and genomes; RF, random forest; SVM, support vector machine; XGBoost, extreme gradient boosting; GLM, generalized linear model.

The rapid development of high-throughput sequencing technologies has led to an exponential growth in the amount of data in the biomedical field. The inherent complexity of biological data has encouraged the increasing use of machine learning in biology to analyze information and predictive models of the underlying biological processes of disease (Auslander et al., 2021; Greener et al., 2022). This study integrated AS and AF transcriptional data from public databases and obtained common genes significantly associated with the diseases by weighted gene co-expression network analysis (WGCNA). Furthermore, a protein-protein interaction (PPI) network was constructed, and enrichment scores of Gene Ontology (GO) and Kyoto Encyclopedia of Genes and Genomes (KEGG) pathway analysis were calculated in order to reveal the common biological mechanisms underlying the comorbidity of AS and AF. Three machine learning methods, named Random Forest (RF), Support Vector Machine (SVM) and Extreme Gradient Boosting (XGBoost), were employed to identify key mitochondrial genes, with the objective of further analyzing disease-related mitochondrial dysfunction mechanisms. The expression levels of the mitochondrial genes identified by the machine learning approaches were then validated using other GEO datasets. ROC curves were plotted in the GEO dataset to assess their predictive value. Additionally, the infiltration of immune cells in the AS and AF dataset was examined. Figure 1 depicts the study’s flow chart.

## 2 Materials and methods

### 2.1 Data sources

Our data were obtained from the Gene Expression Omnibus (GEO) database (Barrett et al., 2013) (http://www.ncbi.nlm.nih.gov/geo/), and we filtered for suitable gene expression data by defining the keywords: “atherosclerosis” or “AS” and “atrial fibrillation” or “AF” with the filter criteria “*Homo sapiens*”. Finally, five datasets were selected. In the AS group, bulk transcriptome data GSE100927 and GSE28829 and single cell transcriptome data GSE253903 were selected, and in the AF group, bulk transcriptome data GSE79768 and GSE41177. The specific sample composition information is shown in Supplementary Table 2. GSE100927 and GSE79768 are used as the training set and GSE28829, GSE41177 and GSE253903 as the external validation set.

### 2.2 Weighted gene co-expression network analysis

WCGNA analysis was performed on GSE100927 and GSE79768 to identify modules associated with AS and AF using the “WGCNA” package (Langfelder and Horvath, 2008) in R, respectively. Genes were ranked based on the standard deviation of their expression, and the top 20% of genes with the greatest variation were selected for inclusion in the following WCGNA analyses. Samples were clustered using hierarchical clustering to identify abnormally outlying samples, and outlying samples were excluded. An automated network and detection module was constructed, and the first soft threshold of 0.9 was selected for the scale-free topological fit index. Next, the gene co-expression network was constructed to measure the degree of co-expression between gene pairs by calculating the correlation between genes using Pearson correlation. Again, hierarchical clustering identified gene co-expression modules, with genes within each module closely related. Finally, module eigenvalues and correlations between calculated module eigenvalues and clinical features were calculated. Module genes closely related to diseases were obtained according to the criteria of correlation coefficient greater than 0.5 and p-value less than 0.05 for subsequent analyses.

### 2.3 Identification of overlapping genes and functional enrichment analysis

The genes in the gene modules with strong disease associations identified by WGCNA analysis in GSE100927 and GSE79768 were taken to intersect in order to identify the overlapping genes associated with disease in both AS and AF. The overlapping genes were visualized using Wayne diagrams. To identify common biological processes and functions in the two diseases, Gene ontology (GO) analysis and Kyoto Encyclopedia of Genes and Genomes (KEGG) pathway enrichment analysis were performed on these genes using the “clusterProfiler” R package (Yu et al., 2012). The GO enrichment analysis included three main functions: biological process (BP), molecular function (MF), and cellular component (CC) (2021). Items or pathways with p value <0.05 were considered significantly enriched and the results were visualized using the “ggplot2″ R package. In parallel, Gene Set Enrichment Analysis (GSEA) was conducted on the GSE100927 and GSE79768 datasets, utilizing the reference dataset “c2. cp.kegg_legacy.v2023.2. Hs.symbols.gmt” from the MSigDB database (Subramanian et al., 2005). With the objective of comprehensively analyzing the key pathways associated with the development of atherosclerosis and key pathways associated with the development of atrial fibrillation. Significantly enriched pathways were identified using screening criteria of P < 0.05 and FDR <0.25, and visualized using the “enrichplot” R package.

### 2.4 Identification of hub genes and construction of protein interaction networks

In order to construct a protein-protein interaction (PPI) network, the overlapping genes identified in the preceding analysis were uploaded to the STRING database (Szklarczyk et al., 2021) (https://cn.string-db.org/), with a minimum required interaction score set to medium confidence (0.4). The species was restricted to human, and the results were visualized using Cytoscape software (Shannon et al., 2003). The hub genes were identified by four algorithms of the CytoHubba plugin (Chin et al., 2014). The following metrics were considered: MMC(Maximum Matching Coefficient), MNC(Maximum Neighbourhood Component), degree, and EPC(Edge Percolated Component). Furthermore, the GeneMANIA database (Franz et al., 2018) (https://genemania.org/) was utilized to analyze gene network of the hub genes.

### 2.5 Identification of mitochondrial related hub genes based on machine learning

A collection of mitochondrial genes was obtained from the mitochondrial protein database MitoCarta 3.0 (Rath et al., 2021) (https://www.broadinstitute.org/mitocarta). The intersection of these mitochondrial genes with common genes associated with the two diseases was analyzed using a Wayne diagram. In order to identify key mitochondrial genes associated with AF and AS, three machine learning algorithms, including random forest (RF), support vector machine (SVM), and extreme gradient boosting (XGBoost), were employed to further screen mitochondrial genes associated with the two diseases. These three machine learning methods are commonly used to screen genes for features. The training set was divided using the “caTools” R package. In the AS training set, 70% of the samples were utilized to construct the model, with 30% of the samples employed to validate the model. In the AF training set, 60% of the samples were used to construct the model, with 40% of the samples used to validate the model. Three machine learning models were constructed using the “caret” R package with the presence or absence of disease as the dependent variable and common mitochondria-related genes as the input independent variables (Jiang et al., 2022). The remaining samples were employed to assess the performance of the constructed models. Due to the opaque nature of machine learning algorithms, the constructed models must be interpreted in order to facilitate their wider comprehension. The “DALEX” R package is designed for the interpretation and analysis of complex predictive models (Biecek, 2018). This package was employed to elucidate the relationships between the input variables and the output results of the constructed RF, SVM, and XGBoost models. The diagnostic results of the models are calculated and visualized by calculating and visualizing the residuals of the models. The smaller the residuals, the better the diagnostic performance of the model. The significance of the variables was evaluated by calculating the Root Mean Square Error (RMSE) (the extent to which the absence of a characteristic variable affects the predicted value of the response variable), with a larger root mean square error indicating a more crucial variable. The accuracy of the model predictions was validated through the use of the predict function. The performance of the model under different thresholds was visualized by the receiver operating characteristic (ROC) curve (Grau et al., 2015). Additionally, the model was evaluated by the area under the curve (AUC), with an AUC value closer to 1 indicating that the model has a higher true positive rate and a lower one, i.e., the better the model performance. The closer the AUC value is to 1, the higher the true-positive rate and the lower the false-positive rate of the model, indicating a higher level of model performance. Conversely, an AUC value of 0.5 indicates that the model performance is equivalent to random guessing.

### 2.6 Validation of key mitochondria-related genes and assessment of their diagnostic value

The identified key mitochondrial genes were validated in external validation sets of AS and AF, respectively. The key mitochondrial genes were subjected to a Wilcoxon test to ascertain whether there were any significant differences between the normal and disease groups. The results were then plotted in the form of box plots. P-values of less than 0.05 were deemed to indicate a statistically significant difference between the two groups. Subsequently, the ROC curves of the diagnostic value of key mitochondrial genes in the training and validation sets were plotted using the “pROC” R package, and the AUC was calculated to assess the accuracy of the prediction of key mitochondrial genes (Grau et al., 2015).

### 2.7 Immune cell infiltration analysis

CIBERSORT is capable of calculating the proportion of distinct immune cells present in a gene expression profile through the utilization of a deconvolution algorithm (Chen et al., 2018). The AS gene expression matrix (GSE100927) was subjected to analysis for the purpose of determining the extent of immune cell infiltration utilizing the CIBERSORT algorithm. Histograms and violin plots were employed to illustrate the relative proportions and discrepancies in the expression profiles of immune cell types between the control and AS groups, with a p-value of less than 0.05 deemed to be significantly different. Subsequently, Spearman correlation analysis was conducted to assess the interrelationship among the 22 immune cells. Finally, the correlations between common central genes and the degree of immune cell infiltration, and common mitochondrial key genes and the degree of immune cell infiltration were analyzed using Spearman’s algorithm and visualized by heatmaps and scatter plots, respectively, using a p-value of <0.05 as a screening criterion.

### 2.8 Single-cell RNA sequencing data analysis

The GSE253903 atherosclerosis single-cell transcriptome data, comprising barcode data, gene characterisation data and a gene count matrix, were downloaded from the Gene Expression Omnibus database by Cellranger (10X Genomics) preprocessing. The preprocessing and subsequent analysis will be conducted using the “Seurat” R package within the RStudio environment (Gribov et al., 2010). For subsequent analysis, the data were normalised and the top 3,000 highly variable genes were identified using the SCTransform function and eliminate the effects of the cell cycle. After which PCA, cluster analysis, and Uniform Mobility Approximation and Projection (UMAP) dimensionality reduction were conducted using the RunPCA, FindClusters, and RunUMAP functions, respectively. The batch effect is then corrected using the “harmony” package (KORSUNSKY et al., 2019). The resolution of the dimensionality reduction clustering was confirmed by the “clustree” package to achieve more optimal cluster clustering (Li et al., 2023). Subsequently, the cell clusters were visualised using UMAP plots, generated by the DimPlot function. Furthermore, the R package “SingleR” (Aran et al., 2019) was employed for the automatic annotation of cell types, in conjunction with the cellMarker2.0 database (Zhang et al., 2019) for manual validastion and supplementary annotations. Following the aforementioned procedures to ascertain the expression levels of pivotal mitochondrial genes in diverse cell types of AS and to determine whether there are discrepancies in the expression of pivotal mitochondrial genes across distinct subgroups, the “ggplot2” R package was utilized for visualization.

### 2.9 Molecular docking

Small molecule drugs were obtained based on the IC50 values of drugs interacting with MRPS23 and CASP8 using the Drug Sensitivity database GDSC (Genomics of Drug Sensitivity in Cancer). Search for the corresponding protein ids of MRPS23 and CASP8 in the uiniprot database. Download the corresponding 3D structures of the proteins from the PDB database based on these ids, import the structures into pymol, and select the top 4 small molecules corresponding to each gene using the small molecules obtained from the above drug sensitivity analysis. Molecular docking was performed using autodock 4.2, and the results were analyzed using pymol to demonstrate the interlinking of H bonds between receptor-ligands.

### 2.10 Validation of selected DEGs’ transcription in fresh normal, atherosclerosis and atrial fibrillation patients blood sample using quantitative real-time PCR

We analyzed samples from normal, Atherosclerosis and Atrial Fibrillation patients Blood sample. The detailed clinicopathological information for all the enrolled patients was available (Supplementary Table 4). Every specimen was anonymously handled based on ethical standards. All patients provided written informed consent and our study was approved by the hospital’s Ethical Review Committee.

The total RNA was extracted using Trizol reagent and reverse-transcribed into complementary DNA (cDNA) for quantitative real-time polymerase chain reaction (qRT-PCR) following the manufacturer’s instructions. GAPDH gene served as an endogenous control. The primer sequences of selected genes (MRPS23, CASP8) used in the experiment are illustrated in Supplementary Table 3. Each sample was tested in triplicates, and each sample underwent a melting curve analysis to check for the specificity of amplification. The relative expression level was determined as a ratio between the hub genes and the internal control GAPDH in the same mRNA sample, and calculated by the comparative CT method. Levels of hub genes’ expression were analyzed by the 2^−ΔΔCT^ method.

### 2.11 Statistical analysis

Statistical analysis was performed using the bioinformatics tools mentioned above, R Studio software V4.2.1 and GraphPad Prism 6.0. Wilcoxon rank sum test as utilized to assess the statistical significance between the two groups when the data conformed to a normal distribution. Results were considered statistically significant at *P < 0.05, **P < 0.01, ***P < 0.001, and ****P < 0.0001.

## 3 Results

### 3.1 Construction of co-expressed gene modules

**FIGURE 2:**
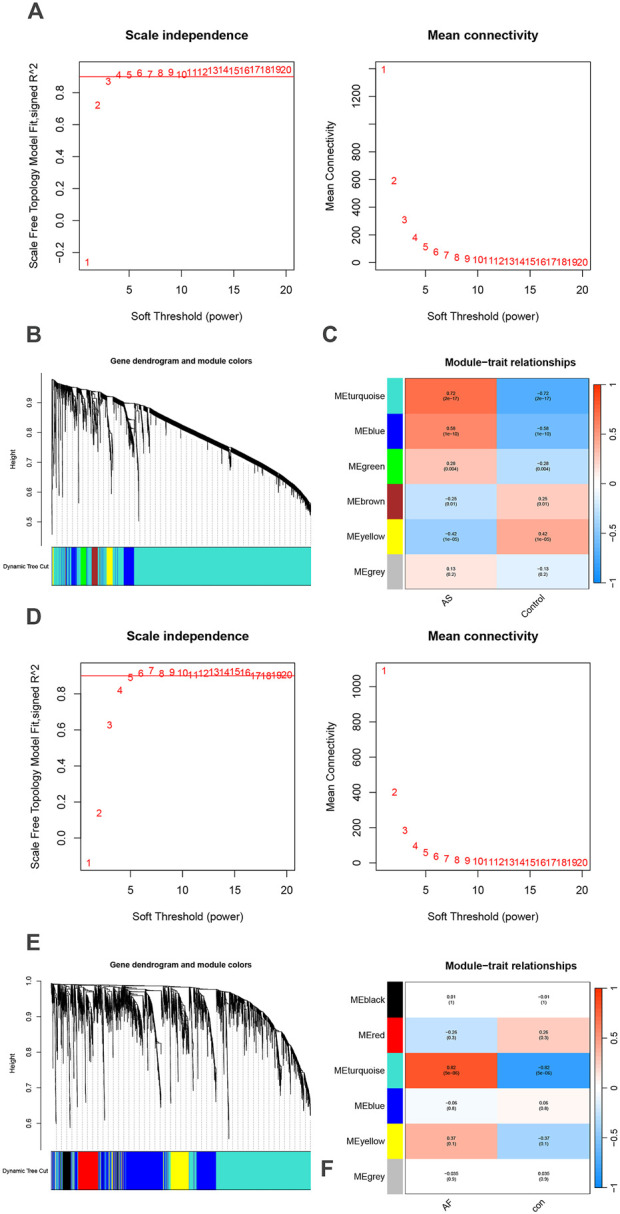
Identification of AS and AF module genes via WGCNA. **(A)** The scale-free fit index for soft-thresholding powers and mean connectivity (GSE100927). **(B)** Hierarchical clustering of genes in AS groups. **(C)** Module-trait relationships heatmap (GSE100927). The colors indicate the strength of the correlation, while the values in parentheses indicate the p-values. **(D)** The scale-free fit index for soft-thresholding powers and mean connectivity (GSE79768). **(E)** Hierarchical clustering of genes in AF groups. **(F)** Module-trait relationships heatmap (GSE79768). The colors indicate the strength of the correlation, while the values in parentheses indicate the p-values.

Modules highly associated with AS and AF and their overlapping genes were identified using WCGNA analysis, respectively. In the AS and AF groups, we determined the proximity matrix weight parameter power values to be 9 and 6, respectively, with the intention of network construction that were more closely aligned with scale-free networks (Figures 2A,D). A TOM matrix gene clustering was constructed based on the weighted correlation coefficients of selected power value pairs (Figures 2B,E). A total of six modules were identified in the AS group (Figure 2C). Among the identified modules, the light blue module (correlation coefficient = 0.72, p-value = 2 × 10^−17^) and the blue module (correlation coefficient = 0.58, p-value = 1 × 10^−10^) exhibited the most significant and positive correlation with AS, containing a total of 4,674 genes. Similarly, in the AF group, six modules were identified. Among the identified modules, the light blue module exhibited the highest correlation coefficient (0.82) and the lowest p-value (5 × 10^−6^) with AS (Figure 2F). This module was also significantly positively associated with AF, containing 2,104 genes. The specific gene information obtained from the WCGNA analysis is presented in Supplementary Tables 1-S1.

### 3.2 Identification of overlapping genes

**FIGURE 3:**
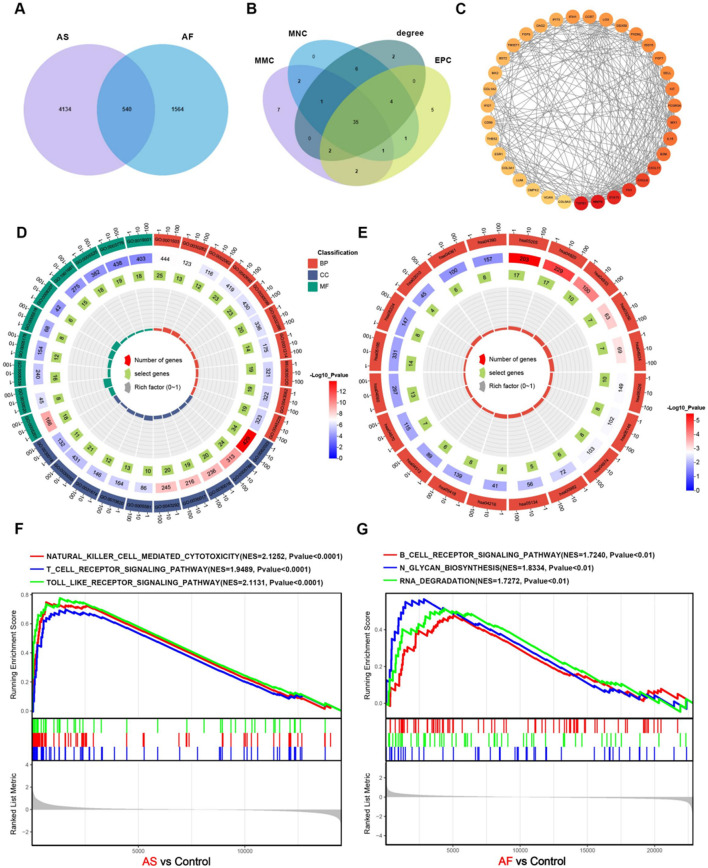
Construction of the core overlapping gene PPI network and enrichment analysis. **(A)** Venn diagram of AS and AF-related module genes. **(B)** Four methods of screening core overlapping genes: MMC, MNC, degree, EPC. **(C)** PPI network of core overlapping genes. **(D)** GO enrichment analysis. The size of the origin represents the number of genes that are enriched in the corresponding pathway. **(E)** KEGG enrichment analysis. The size of the origin represents the number of genes that are enriched in the corresponding pathway. **(F)** GSEA enrichment analysis of upregulated genes in the AS dataset. **(G)** GSEA enrichment analysis of upregulated genes in the AF dataset.

A comparison of the two sets of genes associated with AF and AS revealed 540 genes that were common to both diseases (Figure 3A). Four methods, namely, MMC, MNC, degree, and EPC in CytoHubba, were employed to identify 35 common hub genes in AS and AF (Figure 3B). Subsequently, PPI networks were constructed based on the 540 genes, with the exclusion of those that did not interact. The 35 hub genes were represented according to their degree, with a redder color indicating a greater degree of the node (Figure 3C). This suggests that the gene node may play a more prominent role in the network.

### 3.3 Functional enrichment analysis

To identify common biological processes and functions in the two diseases, Gene ontology (GO) analysis and Kyoto Encyclopedia of Genes and Genomes (KEGG) pathway enrichment analysis were performed on these genes using the “clusterProfiler” R package. The GO enrichment analysis results indicate significant enrichment of biological processes, including muscle cell differentiation, myeloid cell differentiation, regulation of intracellular transport, extracellular matrix organization, and extracellular structure organization. With regard to cellular components, enrichment is observed in collagen-containing extracellular matrix, endoplasmic reticulum lumen, cell-substrate junction, myofibril, and contractile fiber. Moreover, in terms of molecular functions, enrichment is observed in actin binding, extracellular matrix structural constituent, GTP binding, guanyl nucleotide binding, and glycosaminoglycan binding. The findings indicate a potential relationship between the two diseases and cellular metabolism, signaling pathways, immunity, and processes related to the extracellular matrix (Figure 3D; Supplementary Tables 1-S2). The KEGG enrichment analysis results indicate that a significant number of genes are enriched in several pathways, including Cytoskeleton in muscle cells, Proteoglycans in cancer, Cytokine-cytokine receptor interaction, Drug metabolism - cytochrome P450, and Protein digestion and absorption (Figure 3E; Supplementary Tables 1-S3). The results indicate that both diseases may be associated with significant biological processes, including muscle cell morphology maintenance and motor function, immune regulation, inflammatory response, and drug metabolism and clearance. GSEA enrichment analysis revealed that the genes that were upregulated in AS were predominantly enriched in biological pathways, including cytokine-cytokine receptor interaction, B-cell receptor signaling pathway, T-cell receptor signaling pathway, natural killer cell-mediated cytotoxicity, and Toll-like receptor signaling pathway (Figure 3F; Supplementary Tables 1-S4). The genes that were upregulated in AF were primarily enriched in the B-cell receptor signaling pathway, N-glycosylation, spliceosome, RNA degradation, and RNA degradation (Figure 3G; Supplementary Tables 1-S5).

### 3.4 Identification of key mitochondrial genes in AS and AF based on machine learning algorithms

**FIGURE 4:**
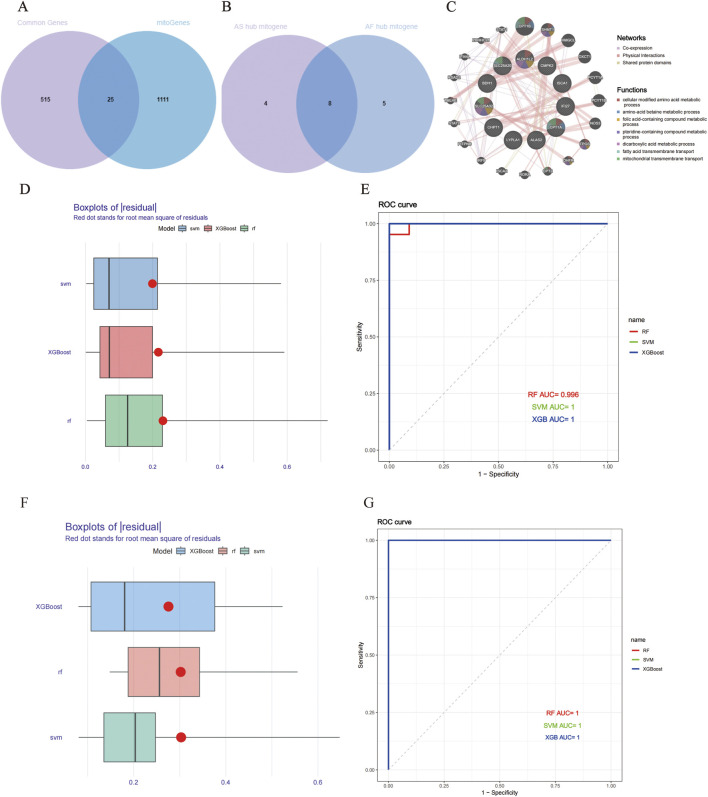
Building and evaluating machine learning models for AS and AF. **(A)** Venn diagram of AS and AF model prediction genes. **(B)** Venn diagram of key mitochondrial genes in AS and AF. **(C)** Co-expression networks of overlapping mitochondrial genes. **(D)** Boxplots of AS residuals. Red dots represent the root mean square of the residuals. **(E)** ROC curve of model prediction accuracy in the AS dataset. **(F)** Boxplots of AF residuals. **(G)** ROC curve of model prediction accuracy in the AF dataset.

In order to illustrate the relationship between mitochondrial function and AS and AF, we obtained 1,136 mitochondria-related genes from the database and took the intersection with the 540 overlapping genes obtained above to get 25 mitochondrial genes related to AS and AF (Figure 4A). Detailed information on overlapping mitochondrial genes is listed in Supplementary Tables 1-S6. The 25 mitochondrial genes and their interactions were predicted, analyzed and visualized through using the GeneMANIA database (Figure 4C). This analysis revealed that the functions of these genes were related to the metabolism process like cellular modified amino acid metabolic process and amino-acid betaine metabolic process. The processes included folic acid-containing compound metabolic process, pteridine-containing compound metabolic process, dicarboxylic acid metabolic process, fatty acid metabolic process, and dicarboxylic acid metabolic process. Additionally, the processes of fatty acid transmembrane transport and mitochondrial transmembrane transport were identified as related. Subsequently, the three machine learning methods, namely, RF, SVM, and XGBoost, were employed to identify the common key mitochondrial genes associated with AF and AS. The training set was divided into a 7:3 ratio for modelling and validation purposes. The performance of the models was evaluated based on the RMSE and ROC curves. The RMSE and ROC curves of the three machine learning models are presented in the form of box plots (Figures 4D–G), with RMSE values around 0.3 and AUC values close to 1. It is evident that these machine learning models, RF, SVM, and XGBoost, all performs excellently. Consequently, in these two datasets, the gene importance scores are evaluated based on RMSE, with the top 15 genes in terms of importance ranking being selected. Finally, we identified 12 key mitochondrial genes in GSE100927 and 13 key mitochondrial genes in GSE79768. After intersecting the two gene sets, we obtained eight crossover genes, including CASP8, CHPT1, CMPK2, CPT1A, MRPS23, NGRN, SLC25A20, and SLC25A3 (Figure 4B; Supplementary Tables 1-S7).

### 3.5 Assessment of predictive value of key mitochondrial genes

**FIGURE 5:**
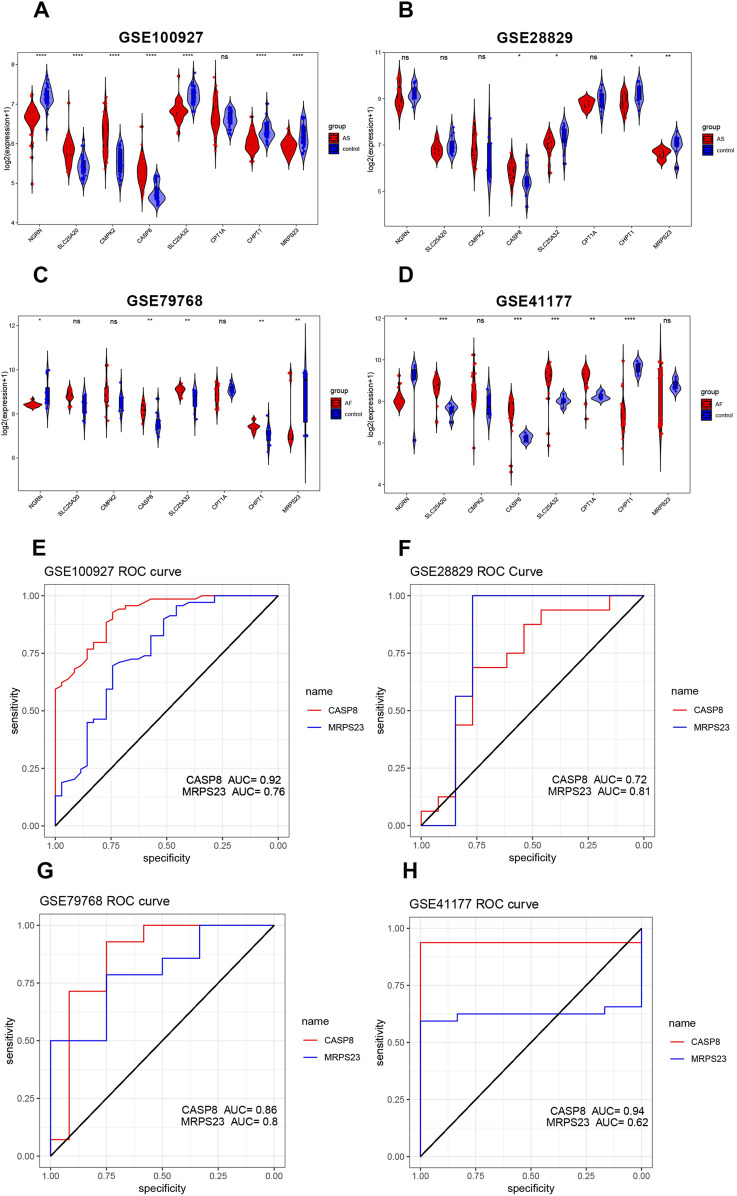
Validation of hub mitochondrial genes and assessment of predictive value. **(A–D)** Validation of the expression levels of six mitochondrial genes in the AS training set (GSE100927), AS validation set (GSE28829), AF training set (GSE79768), and AF validation set (GSE41177). **(E–H)** ROC curves for two key mitochondrial genes (CASP8 and MRPS23) in the AS training set (GSE100927), the AS validation set (GSE28829), the AF training set (GSE79768), and the AF validation set (GSE41177).

The expression differences and trends of the eight key mitochondrial genes were analyzed in the training set (GSE79768, GSE100927) and the test set (GSE41177, GSE28829). The results were presented in boxplots (Figures 5A–D). Finally, CASP8 and MRPS23 were identified as exhibiting significant differences between diseased and normal samples. CASP8 was found to be significantly increased in AS and AF samples. MRPS23 also exhibited a pronounced difference, although not reaching statistical significance in GSE41177. Furthermore, MRPS23 was significantly decreased in AS and AF samples in other datasets. The ROC curves for two genes are shown in Figures 5E–H. In all datasets, the AUC values for CASP8 were greater than 0.7, and those for MRPS23 were greater than 0.7. The differential expression of both genes was validated in the external validation set, and thus these three genes were identified as hub mitochondrial genes that may be associated with AS and AF. This suggests that they may be effective in detecting the combination of AS with AF.

### 3.6 Immune cell infiltration analysis

**FIGURE 6:**
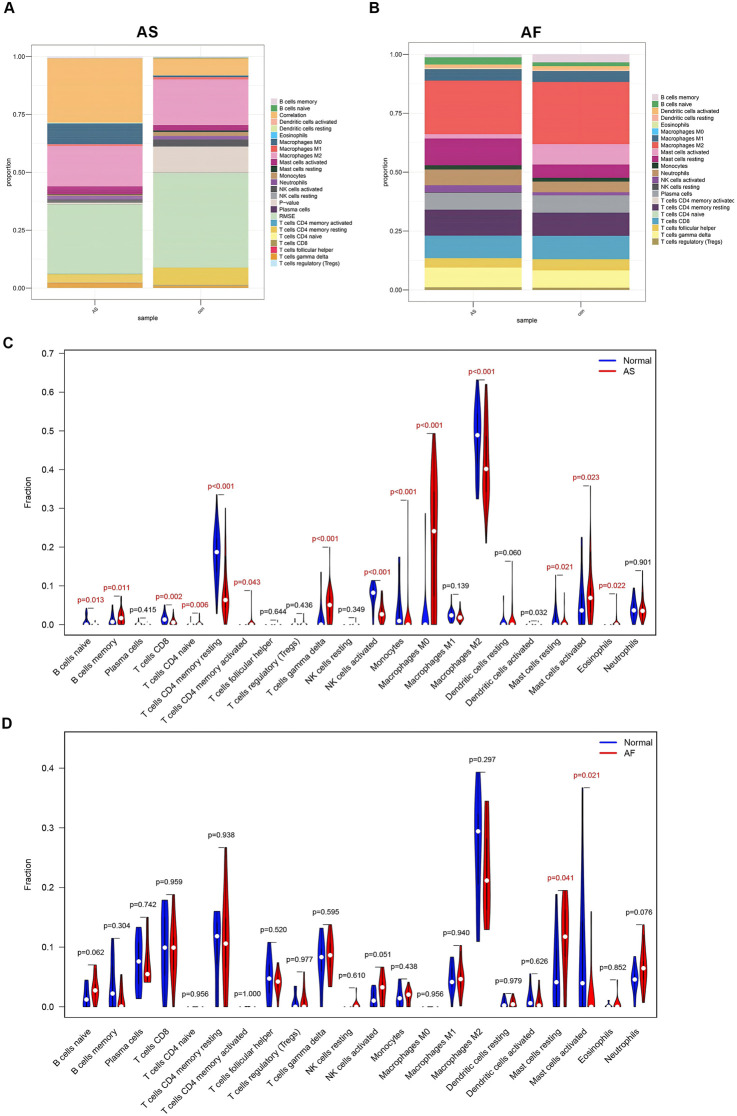
Immune cell infiltration analysis of GSE100927 and GSE79768. **(A)** The proportion of immune cells in different samples of GSE100927. **(B)** The proportion of immune cells in different samples of GSE79768. **(C)** Comparison of immune cell ratios in the AS and control groups. **(D)** Comparison of immune cell ratios in the AF and control groups.

By examining the results of functional enrichment analysis, we discovered that there was a significant enrichment in the process of immune cell production. Additionally, it has been demonstrated that the activation of the immune system and inflammatory response play a significant role in the pathophysiological mechanisms underlying the development of atherosclerosis and atrial fibrillation (Libby et al., 2009; Hu et al., 2015). The “CIBERSORT” algorithm is capable of estimating the proportions of distinct immune cell types present in a gene expression profile by employing a deconvolution algorithm. Consequently, we utilized the “CIBERSORT” algorithm to analyze the gene expression profiles of AS and AF samples, focusing on immune cell infiltration. The detailed results are presented in the Supplementary Tables 1-S8, S9. The proportions of immune cells in the AS and AF groups and their intended counterparts in the control group are shown in Figures 6A,B, respectively. On the one hand, in comparison to the control group, the AS group exhibited elevated levels of B cells memory, T cells gamma delta, macrophages M0, and mast cells activated. Conversely, the AS group exhibited reduced levels of T cells CD8, T cells CD4 memory resting, NK cells activated, monocytes, macrophages M2, mast cells resting, and eosinophils (Figure 6C). On the other hand, the AF group demonstrated elevated levels of mast cells in a resting state, as well as reduced levels of mast cells in an activated state, in comparison to the control group (Figure 6D). The differential expression of immune cell types indicates that each condition may involve unique immune mechanisms and pathways, which could be critical for understanding disease progression and developing targeted therapeutic strategies.

**FIGURE 7:**
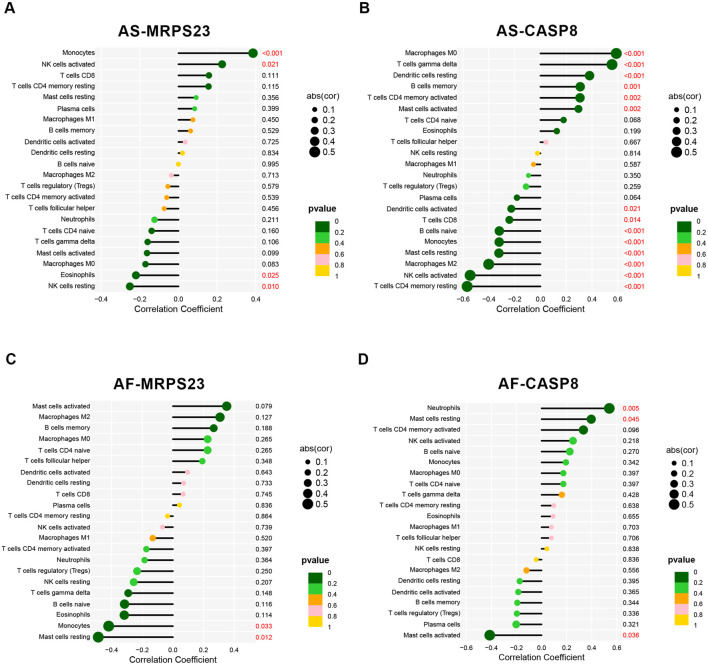
Analysis of key mitochondrial genes and immune cell correlations. **(A)** Lollipop plot of the correlation between MRPS23 gene expression and 22 immune cells in the AS group. **(B)** Lollipop plot of the correlation between CASP8 gene expression and 22 immune cells in the AS group. **(C)** Lollipop plot of the correlation between MRPS23 gene expression and 22 immune cells in the AF group. **(D)** Lollipop plot of the correlation between CASP8 gene expression and 22 immune cells in the AF group.

The correlation between MRPS23 and CASP8 with immune cells in the AS and AF groups was analysed (Supplementary Tables 1-S8). In GSE100927, MRPS23 expression was found to be associated with three immune cell types that exhibited statistically significant differences between the group and its controls (Figure 7A). MRPS23 expression was observed to promote monocyte infiltration (p < 0.001) and NK cell activation (p < 0.05), while simultaneously inhibiting eosinophils (p < 0.05). Concurrently, CASP8 expression was found to correlate with 10 distinct immune cell types (Figure 7B). Notably, a significant positive correlation was observed with M0 macrophages (p < 0.001), T-cell gamma delta (p < 0.001), memory B cells (p < 0.01) and activated mast cells (p < 0.01). The negative correlation between CASP8 expression and the following immune cells was statistically significant: CD4 resting memory T cells (p < 0.001), activated NK cells (p < 0.001), macrophage M2 (p < 0.001), resting mast cells (p < 0.001), monocytes (p < 0.001) and CD8 T cells (p < 0.05). In GSE79768, expression of MRPS23 was found to inhibit resting mast cells (Figure 7C), whereas CASP8 expression correlated with both immune cell types that were statistically different between AF and controls, i.e., CASP8 expression inhibited mast cell activation (Figure 7D). In the context of AS and AF, the expression levels of the MRPS23 and CASP8 genes showed different patterns, and the patterns of immune cell infiltration differed between AS and AF. Correlation analyses between the two genes and immune cell infiltration suggest that the MRPS23 and CASP8 genes may have potential roles in the different immune cell infiltration and regulation.

### 3.7 Validation of MRPS23 and CASP8 genes expression in scRNA-seq data

**FIGURE 8:**
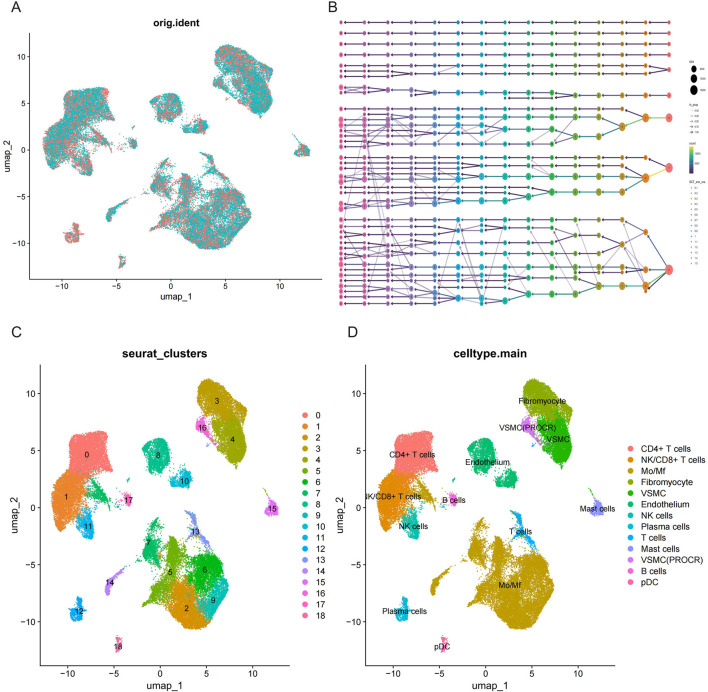
Single cell RNA-seq data preprocessing and cell type annotation. **(A)** UMAP visualisation of cell distribution in AS and control samples. **(B)** Clustree plot for determining resolution (15 resolutions) with principal components (PCs). **(C)** Unified manifold approximation and projection clustering into 18 clusters. **(D)** Cells were annotated using CellMarker and singleR to obtain 13 cell subpopulations.

Single-cell RNA-seq data were obtained from GSE253903, comprising six atherosclerotic samples and six control samples. To guarantee the quality of the data, the following criteria were applied for quality control: filtering of cells with gene counts exceeding 200 and 6,000, mitochondrial gene content exceeding 30%, total UMIs (unique molecular identifiers) exceeding 40,000 and less than 200, erythrocyte content of less than 1%, and gene expression of genes in fewer than three cells (Supplementary Figure S1). In total, the number of cells in the disease and control groups was 22,938 and 29,777, respectively (Figure 8A). Subsequently, batch effects were eliminated by Harmony on the gene expression matrix. The performance of 15 different resolutions was evaluated using the clustree package to identify the optimal resolution for unsupervised clustering (Figure 8B). A resolution of 0.4 was ultimately selected, and the presence of 19 distinct cell clusters was demonstrated using UMAP. Each cluster was delineated with a distinct colour (Figure 8C). Based on the expression pattern of the marker genes and other recognised marker genes. The following genes were used to identify specific cell types: NKG7, KLRB1 (NK cells), TPSAB1, TPSB2 (mast cells), MS4A1, CD79B, CD79A, CD19 (B lymphocyte cells), CD3G, CD3D, CD3E, CD8A (T lymphocyte cells), VWF, ESAM, PECAM1 (endothelial cells), FGF7, COL1A2, DCN (fibromyocyte cells), CD14, CD163, C1QB, C1QA, CD68 (monocyte/macrophage cells), and CNN1, ACTA2, TAGLN, MYL9 (vascular smooth muscle cells). The expression of these marker genes in different cell clusters is shown in Supplementary Figure S2. The results of the clustering obtained by UMAP were further refined and annotated by SingleR and CellMarker2.0 (Figure 8D; Supplementary Tables 1-S11).

### 3.8 Expression patterns of MPRS23 and CASP8 in single cell data

**FIGURE 9:**
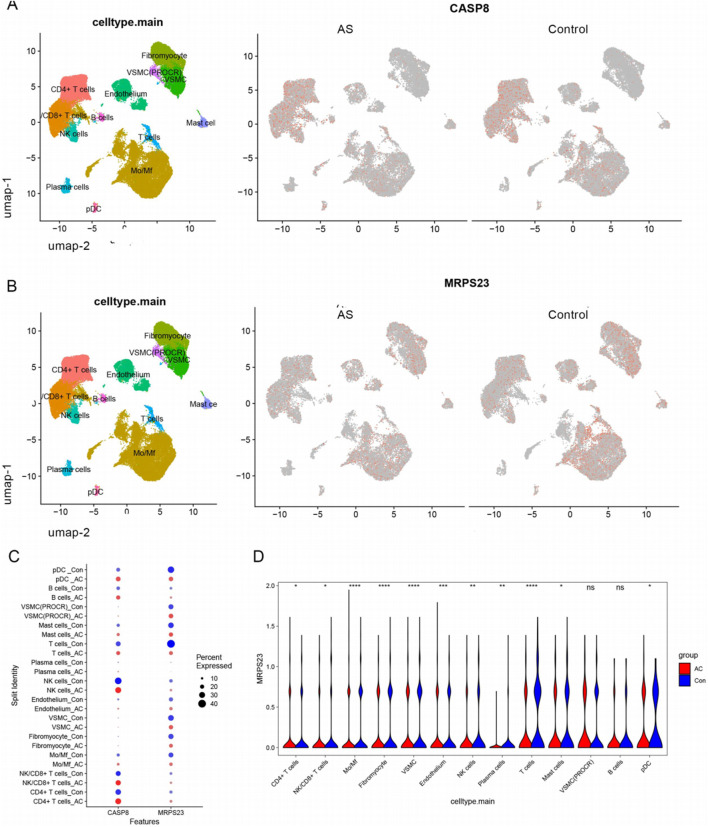
Single-cell analysis of hub mitochondria genes expression profiles. **(A)** FeaturePlots showing the expression pattern of CASP8 in AS and control groups. **(B)** FeaturePlots showing the expression pattern of MRPS23 in AS and control groups. **(C)** Expression dot plots of central mitochondrial genes in the AS and control groups in each cell cluster. **(D)** Violin plots show the differential expression of MRPS23 in each cell population.

The expression patterns of two key mitochondrial genes were described in the intima-media UMAP of atherosclerotic and control samples (Figures 9A,B). MPRS23 expression was found to be relatively downupregulated in the disease group, while CASP8 expression did not differ significantly between the 2 cell clusters. Dot plot analysis revealed that MRPS23 exhibited differential expression between distinct cell clusters in the disease and control groups (Figure 9C). As illustrated in the violin plots, the quantitative analysis of MRPS23 revealed a reduction in various immune and stromal cell types in comparison to the control group, such as T cells, monocytes/macrophages (Mo/Mf), fibroblasts, vascular smooth muscle cells (VSML), natural killer (NK) cells, plasma cells, mast cells, and plasmacytoid dendritic cells (pDC) in comparison to the control group (Figure 9D). This decrease may indicate an immunosuppressive effect of MRPS23, suggesting its potential role in modulating immune responses or its involvement in AS microenvironments. These findings highlight the need for further investigation into the mechanisms underlying these changes and their implications for disease progression and treatment strategies.

### 3.9 Drug sensitivity analysis and molecular docking

**FIGURE 10:**
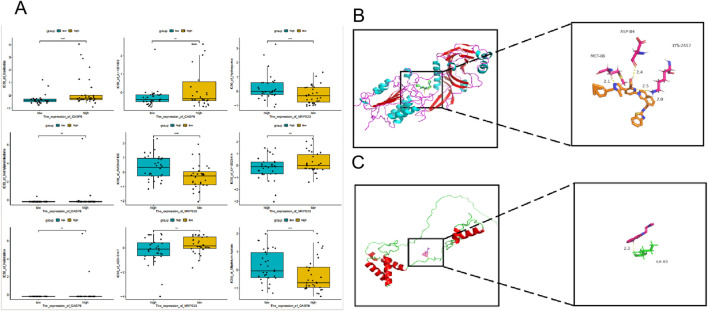
MRPS23 and CASP8 as atherosclerosis drug targets. **(A)** Results of drug sensitivity analysis. **(B)** Molecular docking of MRPS23 with A-1331852. **(C)** Molecular docking of CASP8 with AMONAFIDE.

Based on the results of the drug sensitivity analysis (Figure 10A), in this study, we analyzed the correlations between CASP8 and MRPS23 and A-1331852 as well as AMONAFIDE. Through molecular docking, we found that CASP8 and MRPS23 both showed strong affinity with A-1331852 and AMONAFIDE. The Vina docking score of CASP8 with A-1331852 was −9.71, and the Vina docking score of MRPS23 with AMONAFIDE was −6.49 (Figures 10B,C).

### 3.10 Validation of gene expression

**FIGURE 11:**
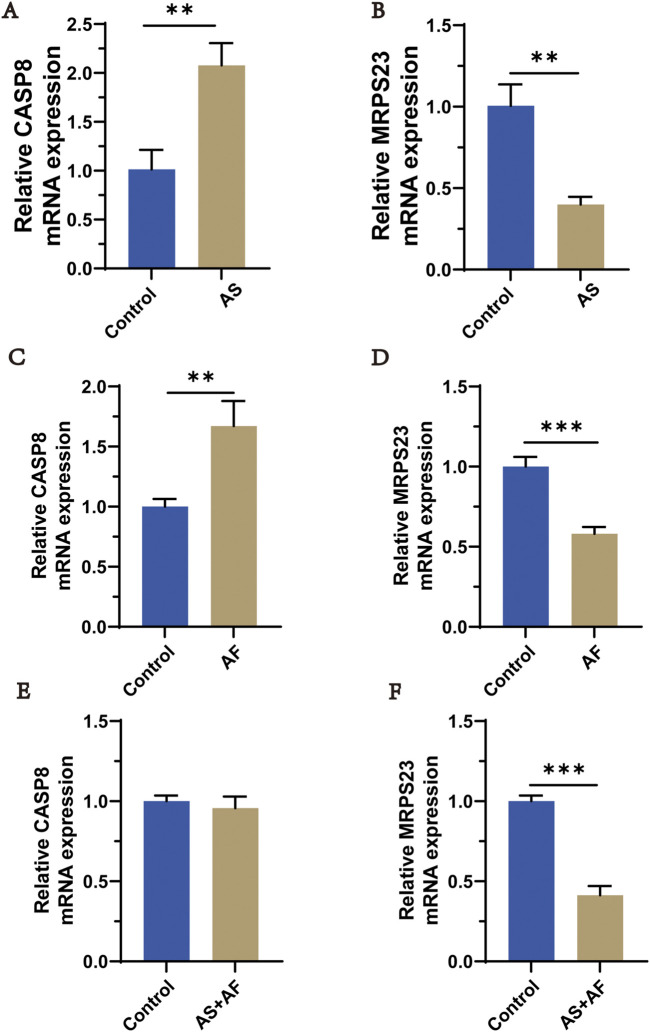
qRT-PCR analysis of hub mitochondria genes expressi in AS and AF patients. **(A)** The expression of CASP8 in AS and control groups. **(B)** The expression of MRPS23 in AS and control groups. **(C)** The expression of CASP8 in AF and control groups. **(D)** The expression of MRPS23 in AF and control groups. **(E)** The expression of CASP8 in AS + AF and control groups. **(F)** The expression of MRPS23 in AS + AF and control groups. (n = 3).

Expression of overlapping genes and FRGs (MRPS23, CASP8) were verified using qRT-PCR in normal, Atherosclerosis and Atrial Fibrillation patients. The qRT-PCR results showed that the levels of CASP8 in the AS group were significantly increased but MRPS23 decreased when compared with the normal group (Figures 11A,B). The trend was similar between the AF group and the normal group (Figures 11C,D). We collected the blood of patients with comorbidities of the two diseases and found that CASP8 expression in the AS + AF group was not significantly different from that in the control group (Figure 11E), but MRPS23 was significantly upregulated in the comorbidities group (Figure 11F), indicating that MRPS23 may be the most potential predictor of comorbidities.

## 4 Discussion

Epidemiological studies have shown that there is a causal association between AS and AF: On one hand, AS and microvascular damage may lead to atrial hypoperfusion and ischemia, which in turn leads to fibrosis and AF. On the other hand, aging of the vasculature, increased pulse and blood pressure, and aortic and peripheral atherosclerosis increase the afterload of the heart during systole and lead to aortic stiffening, a process that in turn results in persistent ventricular and atrial remodeling, ultimately leading to AF (Willeit and Kiechl, 2014; Kristensen et al., 2020; Bekwelem et al., 2018). There are several inflammatory biomarkers identified that are associated with both AS and AF, thus supporting the hypothesis of mutual influence between AS and AF. (Yao et al., 2022; Hu et al., 2015; da Silva, 2017; Abolbashari, 2022).

Mitochondria play multiple roles in the pathogenesis of cardiovascular diseases, from metabolism to signal transduction, to maintaining cellular structure and function. It has been reported that mitochondria contribute to 15% of ATP generation in endothelial cells. Same study found that mitochondrial function plays an important role in AS and AF. Modifications in phospholipids, glucose, and pivotal proteins on the mitochondria-associated endoplasmic reticulum membrane (MAM) have been linked to the advancement of AS (Glanz et al., 2020). And the accumulation of low-density lipoprotein (LDL) in the arterial wall is regarded as a pivotal factor in the pathogenesis of AS and its subsequent progression (Ference et al., 2017). The expression of proteins encoded by the mitochondrial genome is markedly tissue-specific, with the heart displaying a distinctive pattern of mitochondrial metabolic states in comparison to other tissues (Zheng et al., 2017). It seems plausible to suggest that some mitochondrial genes may be associated with the development of cardiovascular disease (Gopisetty and Thangarajan, 2016).

In this study, the 540 genes identified by WGCNA as being co-expressed play a pivotal role in a number of biological processes, including cellular differentiation, immune response and intracellular regulation. In particular, they are involved in muscle cell and myeloid cell differentiation, intracellular transport and GTP binding. The results of the GSEA enrichment analysis also indicate a close link between the two diseases and immune system-related pathways or functions. This is consistent with previous results (Saigusa et al., 2020; Tousoulis et al., 2016; Sage et al., 2019). Therefore we proposed that mitochondrial dysfunction associated with cellular metabolism, immune regulation and inflammatory response may represent a key mechanism in both diseases, as confirmed by the findings of Dumont et al. in AS (Dumont et al., 2021).

Meanwhile, the analysis of immune cell infiltration in the context of AF suggests that mast cells play an important role in the pathogenesis of AF. Mast cells may be significantly increased in number and activity in patients with AF (Hu et al., 2015; Liao et al., 2010). They may play an important role in the onset, maintenance, and recurrence of AF through the promotion of a local inflammatory response, as well as through their effects on atrial electrophysiology and structure. In contrast, AS has been linked to a range of immune cells, including M0 macrophages, mast cells, B cells and T cells. Our results are consistent with the findings from Tay’s research (Tay et al., 2019).

Based on the above results, we performed machine learning methods to screen eight key mitochondrial genes (CASP8, CHPT1, CMPK2, CPT1A, MRPS23, NGRN, SLC25A20 and SLC25A32), in which CASP8 expressed upregulated significantly and MRPS23 expressed downregulated significantly compared with normal group in all datasets. CASP8, as the initiator caspase in the death-receptor pathway, was closely related to AS and AF:upregulated CASP8 could increase the recurrence risk of atrial fibrillation, and activating CASP8 gene in smooth muscle cell in apoptosis processes could influence the progression of AS (Charitakis et al., 2019; Xue et al., 2024; Li et al., 2002; Carlotti et al., 2005). MRPS23 is a component of the small subunit of mitochondrial ribosomes, which are involved in mitochondrial protein synthesis and energy metabolism. Research suggests that MRPS23 may play an important role in the development of cardiovascular disease (Huang et al., 2020; Ittiwut et al., 2023; Zheng et al., 2017).

We analyzed the immune infiltration correlations of MRPS23 and CASP8 genes in both AS and AF contexts, uncovering distinct immune regulatory roles for each gene. Specifically, MRPS23 downregulation in AS and AF was associated with increased infiltration of monocytes and NK cell activation while inhibiting eosinophils, suggesting a targeted modulation of immune cell types that may promote inflammation and immune cell migration. In contrast, CASP8 upregulation correlated with a broader spectrum of immune cells, including a positive association with pro-inflammatory cells such as M0 macrophages, gamma delta T cells, and memory B cells, which are key in chronic inflammatory responses. CASP8 also demonstrated significant negative correlations with immune cells linked to adaptive immunity, such as resting memory CD4 T cells, activated NK cells, and CD8 T cells, suggesting a dual regulatory role that balances immune activation and suppression to prevent excessive inflammation and tissue damage. In the AF-specific context, MRPS23 inhibited resting mast cells, while CASP8 restricted mast cell activation, indicating that both genes might play protective roles against excessive fibrosis and structural remodeling in atrial tissue.

A single-cell transcriptomic dataset for atherosclerosis was subjected to analysis, with a view to identifying pivotal mitochondrial gene expression profiles. Comprehensive intercellular communication network analysis identified myofibroblasts, endothelial cells, M0/Mf macrophages, and vascular smooth muscle cells as key signaling hubs, exhibiting pronounced crosstalk with multiple cellular subsets (Supplementary Figure S3). The results revealed that only MRPS23 was significantly downregulated in the AS group, which is consistent with the trend observed in the bulk transcriptomic analysis. The quantitative analysis of MRPS23 demonstrated a notable reduction in several immune and stromal cell types, including T cells, monocytes/macrophages, fibroblasts, vascular smooth muscle cells, natural killer cells, plasma cells, mast cells, and plasmacytoid dendritic cells, when compared to the control group. These findings suggest a potential immunosuppressive role of MRPS23 within the atherosclerotic environment, which could contribute to the progression of the disease by impairing normal immune responses and promoting inflammation.

Mitochondrial-immune cross-interference has also been reported in myocardial injuries such as diabetic cardiomyopathy (DCM). In DCM, the most significant metabolic disorders in myocardial tissue are decreased glucose utilization and increased fatty acid oxidation. This study uniquely identified that MRPS23-driven mitochondrial ribosomal dysfunction is the common mechanism connecting the immune dysregulation of complications such as and AF., The following are the potential clinical validation and transformation approaches based on current bioinformatics discoveries: 1) Constructing an MRPS23 conditional knockout animal model to simulate the comorbbiosis phenotype of AS/AF and evaluate the reversibility of its metabolic-immunophenotype; 2) Conduct a multicenter cohort study to verify the correlation between the expression dynamics of MRPS23 and disease progression; 3) Explore the possibility of epigenetic regulation of MRPS23 (such as methylation modification) as a non-invasive biomarker.

## 5 Conclusion

By applying machine learning algorithms for screening and validation across multiple datasets, the MRPS23 gene was identified as a potential biomarker for AS binding to AF. In addition, MRPS23 was found to be significantly associated with a variety of immune cells. This provides new insights into the underlying biological mechanisms of AS with AF, which will help to discover and obtain new therapeutic targets.

## Data Availability

The original contributions presented in the study are included in the article/Supplementary Material, further inquiries can be directed to the corresponding author.
