# Supplementary material for: Comprehensive bioinformatics analysis of the common mechanism of atherosclerosis and atrial fibrillation: emphasizing mitochondrial metabolic disorder and immune inflammation
Source: Front Mol Biosci. 2025 Jun 18;12:1595048. doi: 10.3389/fmolb.2025.1595048 (PMC12215116; doi:10.3389/fmolb.2025.1595048)

**Supplementary Figure 1:** Distribution of gene counts, UMIs, mitochondrial gene content and erythrocyte content before and after quality control.

**Before**


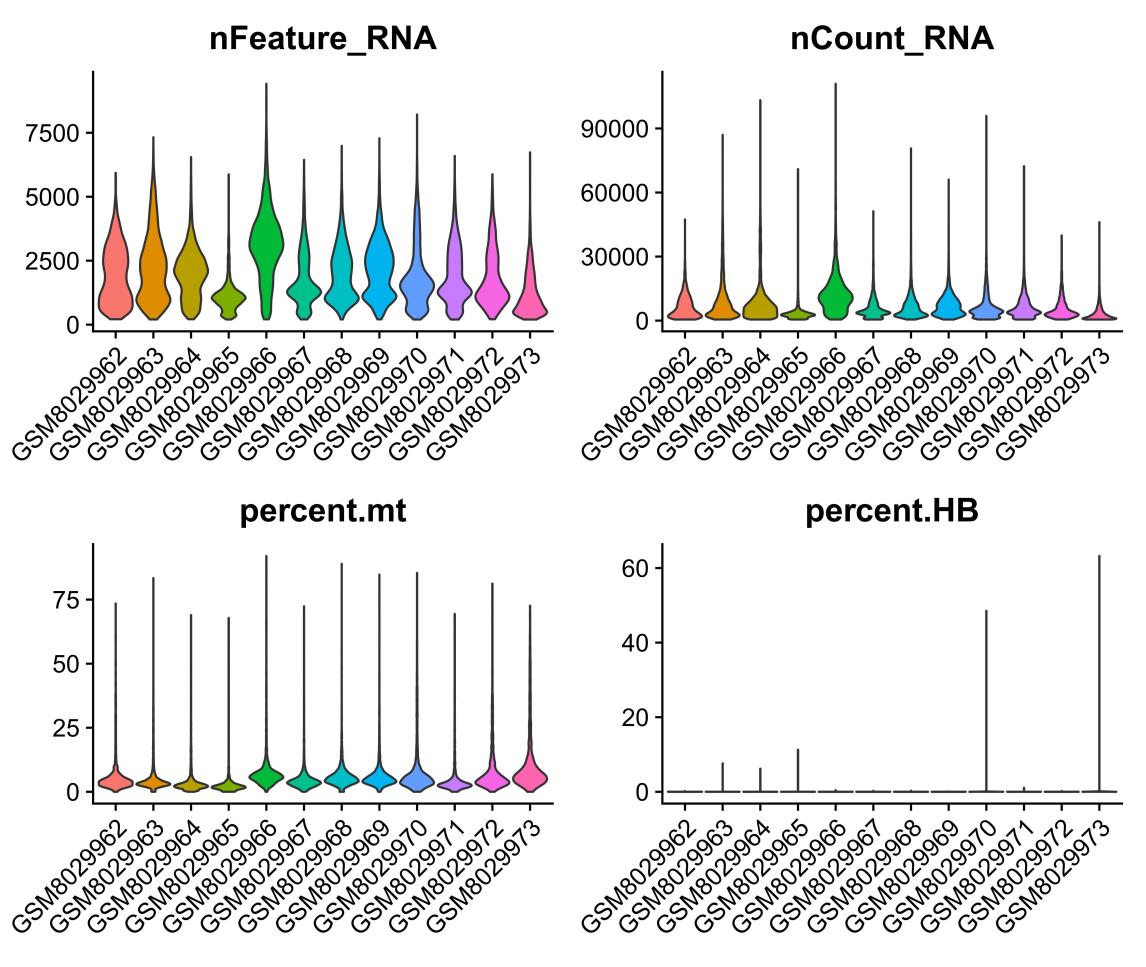


**After**


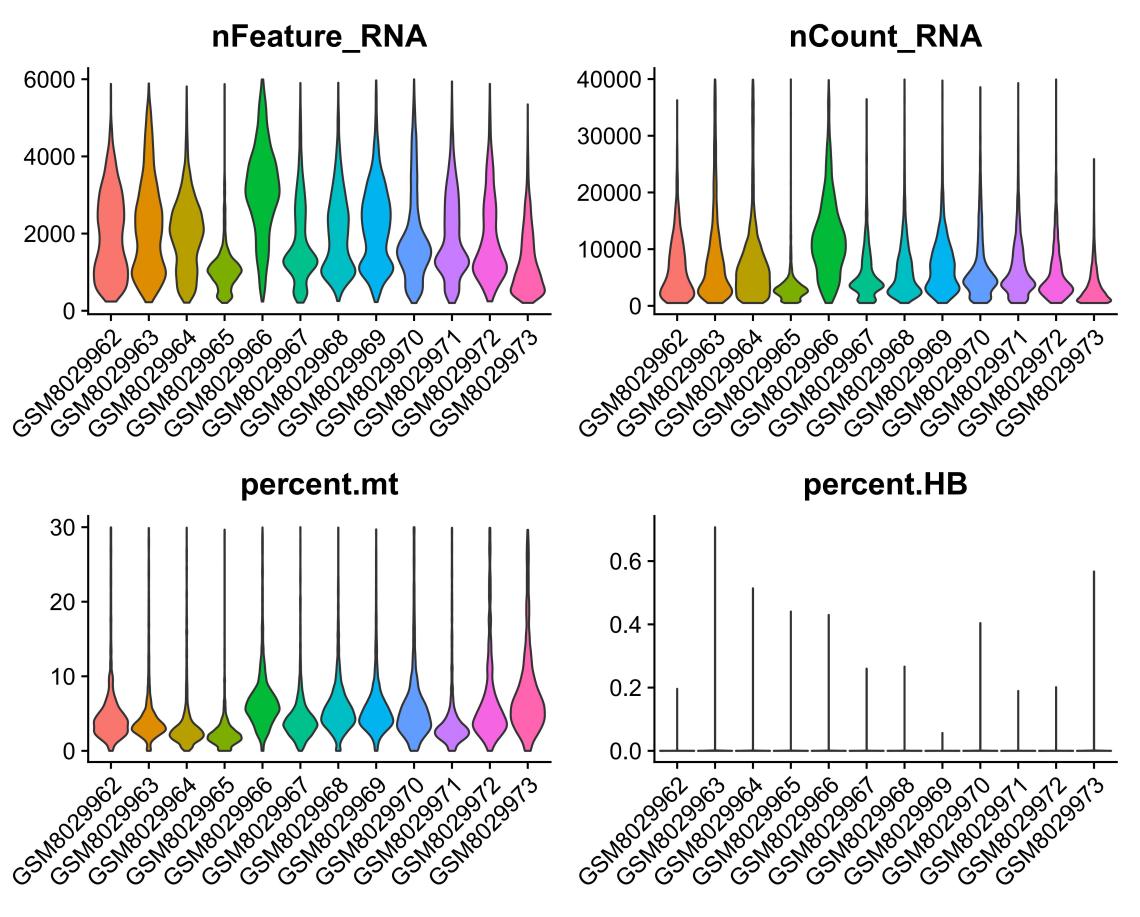


**Supplementary Figure 2:** Expression of marker genes in different cell clusters.


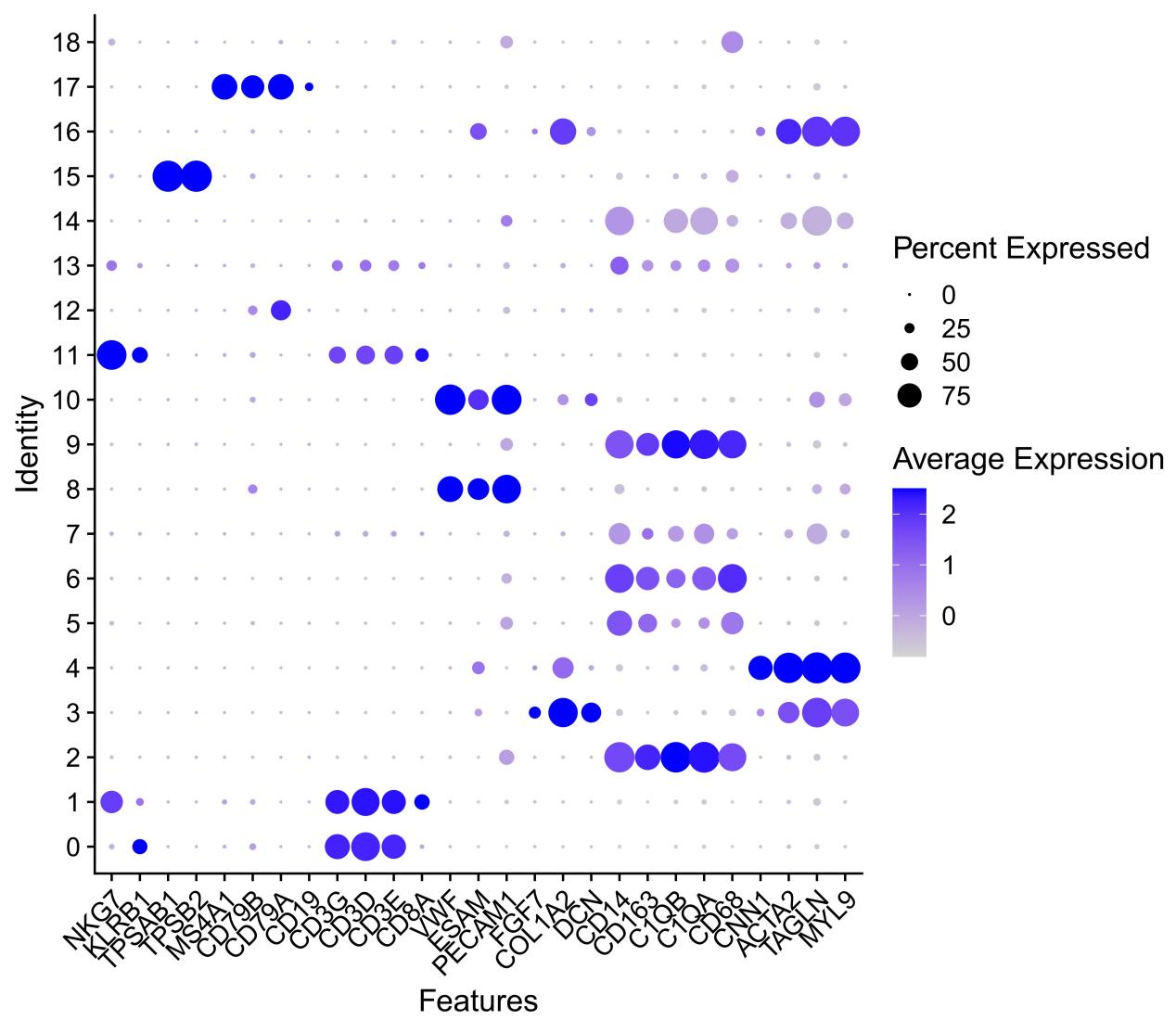


**Supplementary Figure 3** Comprehensive intercellular communication network analysis.


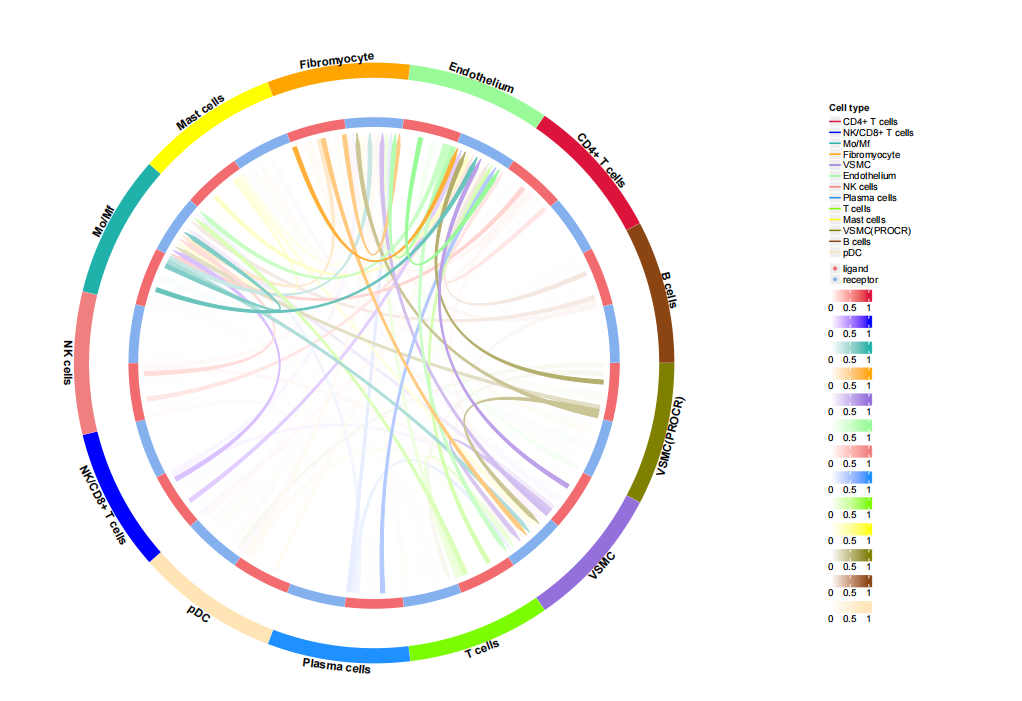

Supplement: Supplementary file 1 [file Supplementaryfile1.zip › The supplementary materials/Supplementary Figure.docx]
