# Supplementary material for: Comprehensive bioinformatics analysis of the common mechanism of atherosclerosis and atrial fibrillation: emphasizing mitochondrial metabolic disorder and immune inflammation
Source: Front Mol Biosci. 2025 Jun 18;12:1595048. doi: 10.3389/fmolb.2025.1595048 (PMC12215116; doi:10.3389/fmolb.2025.1595048)
Supplement: Supplementary file 1 [file Supplementaryfile1.zip › The supplementary materials/Supplementary Table2.docx]

Table 1. Sample composition information

| Data set | Sample composition | Tissue | Sequencing platform |
| --- | --- | --- | --- |
| GSE100927 | 69 AS patients  35 control patients | Carotid, femoral and infra-popliteal arteries | [GPL17077](https://www.ncbi.nlm.nih.gov/geo/query/acc.cgi?acc=GPL17077) |
| GSE28829 | 16 AS patients  13 control patients | Atherosclerotic carotid artery segments | [GPL570](https://www.ncbi.nlm.nih.gov/geo/query/acc.cgi?acc=GPL570) |
| GSE79768 | 14 AF patients  12 control patients | Left atrial and right atrial | [GPL570](https://www.ncbi.nlm.nih.gov/geo/query/acc.cgi?acc=GPL570) |
| GSE41177 | 6 AF patients  32 control patients | LA-PV junction and left atrial appendage | [GPL570](https://www.ncbi.nlm.nih.gov/geo/query/acc.cgi?acc=GPL570) |
| GSE253903 | 6 AS patients  6 control | carotid athersclerotic plaques | GPL24676 |

The datasets were acquired from the GEO database (https://www.ncbi.nlm.nih.gov/geo/).
