# Supplementary material for: Comprehensive bioinformatics analysis of the common mechanism of atherosclerosis and atrial fibrillation: emphasizing mitochondrial metabolic disorder and immune inflammation
Source: Front Mol Biosci. 2025 Jun 18;12:1595048. doi: 10.3389/fmolb.2025.1595048 (PMC12215116; doi:10.3389/fmolb.2025.1595048)
Supplement: Supplementary file 1 [file Supplementaryfile1.zip › The supplementary materials/Supplementary Table3.docx]

Supplementary table 2.

Primer sequences of PCR

| Primers | Sequence (5’→3’) | |
| --- | --- | --- |
| CASP8(178bp) | Forward | CTGCCTACAGGGTCATGCTC |
|  | Reverse | TCCAACTTTCCTTCTCCCAGG |
| MRPS23(109bp) | Forward | GGAGGTAGTCACGTTTCCCG |
|  | Reverse | CCTCCAAATGCTGGTCCTGT |
| GAPDH(168bp) | Forward | ATGGGCAGCCGTTAGGAAA |
|  | Reverse | CGCCCAATACGACCAAATC |
